# Prognostic Significance of the CALM Index in Surgically Resected Non-Small Cell Lung Cancer

**DOI:** 10.3390/jcm15155772

**Published:** 2026-07-23

**Authors:** Soomin An, Wankyu Eo, Sookyung Lee, Dae Hyun Kim

**Affiliations:** 1 Department of Nursing, Dongyang University, Yeongju 36040, Gyeongbuk, Republic of Korea; sue339@naver.com; 2 College of Medicine, Kyung Hee University, Seoul 05278, Republic of Korea; 3 Department of Clinical Oncology, College of Korean Medicine, Kyung Hee University, Seoul 05278, Republic of Korea; 4 Department of Thoracic Surgery, Kyung Hee University Hospital at Gangdong, Seoul 05278, Republic of Korea

**Keywords:** immunity, non-small cell lung cancer, nutrition, prognosis, risk stratification

## Abstract

**Background**: Anatomical staging is often insufficient for predicting outcomes in stage I-IIIA non-small cell lung cancer (NSCLC) patients after curative resection due to inherent prognostic variability. In this study, we evaluated the prognostic value of the C-reactive protein–albumin–lymphocyte–monocyte (CALM) index as an integrative biomarker for overall survival (OS). **Methods**: This retrospective study included 533 patients with stage I-IIIA NSCLC. We assessed the association between the CALM index and OS using multivariable Cox proportional hazards models, adjusted for established clinicopathological factors. We employed LASSO-penalized regression to optimize variable selection and ensure model stability. Comprehensive performance metrics were calculated, including Harrell’s C-index, integrated area under the curve (iAUC), integrated discrimination improvement (IDI), and decision-curve analysis (DCA). **Results**: The CALM index was identified as a stable, independent predictor of OS across both conventional and LASSO-penalized models. The final multivariable model incorporated the CALM index alongside age, pathological stage, American Society of Anesthesiologists status, pleural invasion, and the modified Shine–Lal index. Incorporation of the CALM index significantly enhanced model discrimination and risk reclassification at 3 and 5 years, while consistently increasing net clinical benefit across a wide range of threshold probabilities. **Conclusions**: The CALM index is a biologically plausible, robust, and readily accessible prognostic biomarker that provides incremental prognostic information beyond established factors in patients with resected stage I-IIIA NSCLC. Its integration into postoperative assessment offers a framework for refined prognostic stratification and more individualized clinical decision-making.

## 1. Introduction

Despite advancements in treatment, lung cancer remains a primary contributor to global cancer-related mortality [1], with non-small cell lung cancer (NSCLC) constituting the vast majority of diagnoses [2]. While the tumor–node–metastasis (TNM) classification has remained the cornerstone of prognostic evaluation and therapeutic decision-making since its inception, anatomical staging often fails to capture the full spectrum of disease biology [3]. Marked heterogeneity in clinical outcomes among patients with identical pathological stages underscores the limitations of stage-based systems in accounting for occult residual disease or aggressive tumor biology following resection. Consequently, recurrence and mortality rates remain substantial even in early-stage NSCLC [4,5,6].

While established clinicopathological indicators—such as patient age, smoking status, pleural invasion (PL), and tumor size—are standard for risk assessment, they primarily reflect local tumor extent rather than the systemic tumor–host interactions that critically influence recurrence and survival [5,7,8,9,10,11,12,13,14,15]. These limitations have spurred the search for practical, blood-based biomarkers that complement conventional staging to facilitate personalized prognostic assessment. Routine laboratory markers are particularly attractive due to their cost-effectiveness, reproducibility, and widespread accessibility.

Key serum and cellular markers have been individually linked to NSCLC prognosis: C-reactive protein (CRP) reflects systemic inflammation, serum albumin indicates nutritional status, absolute lymphocyte count (ALC) denotes adaptive immune function, and absolute monocyte count (AMC) serves as a marker for tumor-promoting inflammation and angiogenesis [13,16,17,18,19,20,21,22]. Because cancer progression is driven by the synergy between nutritional reserves, inflammation, and immune competence, indices that integrate these distinct domains may provide superior prognostic resolution compared to single-parameter metrics.

The recently introduced CRP–albumin–lymphocyte (CALLY) index has demonstrated prognostic relevance in heterogeneous NSCLC populations [7]. We expanded upon this framework by developing the CRP–albumin–lymphocyte–monocyte (CALM) index. By incorporating AMC, the CALM index captures critical, complementary dimensions—specifically myeloid-driven immunosuppression and tumor-promoting inflammation—not fully represented by the other constituents [23,24]. By synthesizing inflammatory, nutritional, lymphoid, and myeloid domains, the CALM index offers a more comprehensive assessment of the systemic milieu relevant to postoperative outcomes.

Accordingly, this study evaluated the independent prognostic significance, comparative performance, and clinical utility of the CALM index in predicting overall survival (OS) in a cohort of 533 patients with stage I–IIIA NSCLC who underwent curative resection. We hypothesized that the CALM index would outperform established composite biomarkers and provide prognostic information independent of conventional clinicopathological factors.

## 2. Materials and Methods

### 2.1. Patients

This retrospective analysis evaluated 533 consecutive patients diagnosed with NSCLC who underwent curative surgical resection at Kyung Hee University Hospital at Gangdong (Seoul, Republic of Korea) from January 2009 to October 2024. Our institutional protocol for preoperative staging included contrast-enhanced computed tomography (CT) of the chest and abdominopelvic regions, along with positron emission tomography–computed tomography (PET-CT). Pathological stage classification was based on the 8th edition of the American Joint Committee on Cancer system [6]. The postoperative treatment followed contemporary clinical guidelines. Patients with stage II–IIIA disease received adjuvant platinum-based doublet chemotherapy comprising cisplatin combined with paclitaxel, vinorelbine, or pemetrexed. Adjuvant osimertinib was administered to patients with stage IB disease harboring sensitizing epidermal growth factor receptor mutations (exon 19 deletion or L858R) [25]. Surveillance protocols consisted of regular imaging at 3-to-6-month intervals for the first 3 years, followed by every 6 months and annually thereafter.

Participants were included if they met the following: (1) confirmed stage I–II or selected N2-negative stage IIIA disease [26,27]; (2) complete preoperative imaging; (3) margin-negative (R0) surgical outcomes [28]; (4) baseline data available within 7 days of surgery; and (5) documented survival outcomes. We excluded patients who received neoadjuvant therapy, those with advanced disease stages (N2-positive IIIA, IIIB, or IV), individuals with secondary malignancies within the last 5 years, or patients with active, ongoing systemic infections or connective tissue diseases at the time of resection.

### 2.2. Clinical Characteristics

Clinical and pathological data were retrospectively collected from electronic medical records. The recorded demographic factors included age, sex, smoking status, alcohol intake (defined as consumption of alcohol more than once per week) [29], American Society of Anesthesiologists physical status (ASA-PS), and body mass index. Treatment-related data included surgical technique, extent of resection, and administration of adjuvant therapy after surgery. Tumor variables comprised histology, size, pathological stage, and PL [30]; lymphatic, vascular, and perineural invasion; and the presence of microscopic residual disease.

Baseline laboratory values—collected within one week of surgery—were utilized, prioritizing the measurement closest to the operation date [31,32]. Hematological variables comprised total white blood cell (WBC) count and differential counts, red blood cell (RBC) parameters (including hemoglobin, hematocrit, and erythrocyte indices), and platelet counts. The modified Shine–Lal index (mSLI), a surrogate marker of microcytic erythropoiesis, was calculated using mean corpuscular volume (MCV) and mean corpuscular hemoglobin (MCH) [33]. Biochemical measurements included CRP, serum albumin, total protein, and liver enzymes. The CALM index was calculated as: (*β*1 × CRP) + (*β*2 × albumin) + (*β*3 × ALC) + (*β*4 × AMC), where *β* denotes regression coefficient for each variable.

### 2.3. Statistical Analysis Framework

#### 2.3.1. Definition of Outcome and Data Handling

The OS served as the primary endpoint, calculated from the date of surgery to death or the final follow-up. Patients who were alive at the time of their last follow-up were censored on that date. Continuous covariates were modeled on their original scales without categorization to preserve data granularity and optimize the statistical efficiency. We used a complete-case approach for missing values. Statistical significance was set at a two-sided *p* < 0.05 using R (version 4.4.0).

#### 2.3.2. Cox Regression-Based Survival Modeling

Prognostic associations with OS were first screened using univariate Cox proportional hazards regression. Variables demonstrating statistical significance (*p* < 0.05) in these initial analyses were subsequently entered into multivariable models to evaluate their independent predictive value. To reduce multicollinearity and minimize overfitting, predictor selection was further refined using least absolute shrinkage and selection operator (LASSO)-penalized Cox regression with cross-validated selection of the penalty parameter (λ). Collinearity among candidate covariates was assessed using variance inflation factors. Proportional hazards assumption was formally evaluated for each predictor and variables that violated this assumption were excluded from the multivariable modeling.

To better delineate risk gradients across continuous predictors, log-relative hazard plots were constructed, recognizing that conventional Cox model hazard ratios (HRs) may inadequately capture non-linear or graded associations. HRs for continuous covariates were expressed per unit increment. Furthermore, OS probabilities were estimated using the Kaplan–Meier method, with inter-group differences compared via the log-rank test. Patients were stratified into “Low CALM” and “High CALM” cohorts based on the median value of the index to visualize the prognostic discrimination of the CALM index.

Finally, to determine if the CALM index provided incremental prognostic value beyond its components, we compared nested models using likelihood ratio tests, Harrell’s C-index, and the Akaike information criterion (AIC).

#### 2.3.3. Data-Driven Identification of Determinants of the CALM Index

To elucidate the drivers of the CALM index, we employed a dual-modeling approach. Initially, we used LASSO regression to select key clinical and laboratory predictors. We then used an Extreme Gradient Boosting (XGBoost) model to capture complex, non-linear dependencies. Interpretability was achieved through SHapley Additive exPlanations (SHAP), prioritizing both gain-based importance and mean absolute SHAP values.

#### 2.3.4. Model Performance Assessment and Clinical Utility

To evaluate predictive performance, we compared the Full Model (FM) against two reference frameworks: a Baseline Model (BM) restricted to pathological stage, and an Intermediate Model (IM) encompassing all FM covariates except the CALM index. Discriminative capability was assessed using Harrell’s C-index and integrated area under the curve (iAUC), with internal validation through 1000 bootstrap re-samples. Integrated discrimination improvement (IDI) was assessed for quantifying the performance improvement of the FM over the BM and IM. We examined clinical utility via decision curve analysis (DCA) to determine net benefit across threshold probabilities [34]. Furthermore, we benchmarked the CALM index’s prognostic contribution against established inflammatory and nutritional markers, specifically the CALLY index, hemoglobin–albumin–lymphocyte–platelet (HALP) score, systemic immune-inflammation index (SII), and systemic inflammation response index (SIRI) [7,15,35,36,37,38,39]. Finally, we visualized the prognostic model using a nomogram and assessed calibration accuracy through bootstrap-corrected calibration plots [34].

## 3. Results

### 3.1. Baseline Clinicopathological and Laboratory Characteristics

The study cohort comprised 533 patients who underwent curative-intent resection for NSCLC. The population was ethnically homogeneous, with the vast majority identified as East Asians (98.1%, *n* = 523) and a minority identified as Caucasians (1.9%, *n* = 10). Based on postoperative pathological staging, most patients were classified as stage I (*n* = 389, 73.0%), followed by stage II (*n* = 78, 14.6%), and stage IIIA disease (*n* = 66, 12.4%) (Table 1). The CALM index was calculated using the following weighted linear combination of inflammatory and nutritional biomarkers: the CALM index = (0.015 × CRP [mg/L]) − (1.836 × albumin [g/dL]) − (0.49 × ALC [10^3^/μL]) + (2.05 × AMC [10^3^/μL]). The weighting coefficients were derived from Cox proportional hazards modeling, reflecting the relative contribution of each component to OS risk.

### 3.2. Identification and Validation of Independent Predictors of Overall Survival

Univariate Cox regression identified a wide array of demographic, clinical, and laboratory variables significantly linked to OS. These factors encompassed age, sex, smoking status, ASA-PS, tumor characteristics (histological subtype, size, PL, invasion status, and pathological stage), surgical technique, and various hematological and biochemical markers, including the CALM index.

**Figure 1:**
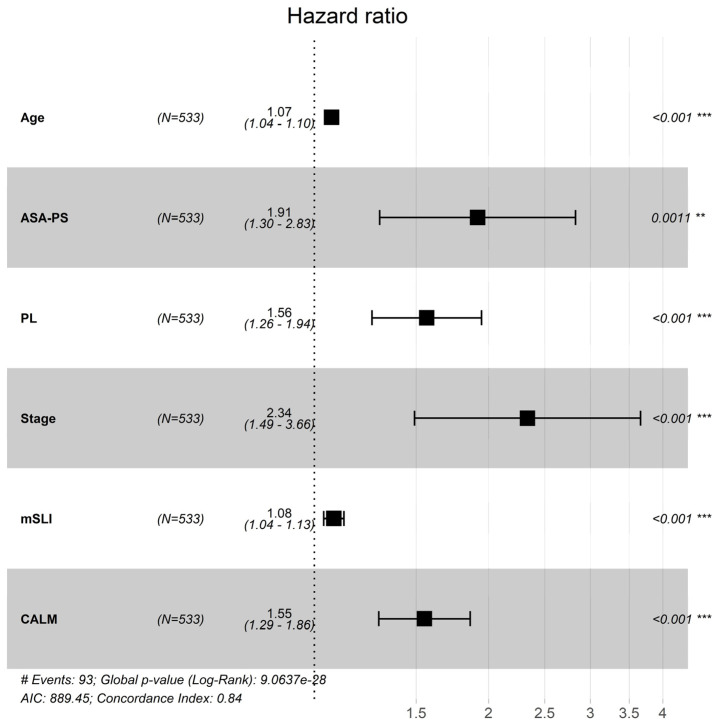
Cox regression and penalized variable selection for overall survival. PL, pleural invasion; CALM, CRP–albumin–lymphocyte–monocyte index. Asterisks indicate statistical significance: ** *p* < 0.01 and *** *p* < 0.001.

Following multivariable Cox proportional hazards modeling, independent prognostic factors for OS included advanced age (HR 1.07), higher ASA-PS (HR 1.91), presence of pleural invasion (HR 1.56), more advanced pathological stage (HR 2.34), higher mSLI (HR 1.08), and an elevated CALM index (HR 1.55); all parameters reached statistical significance (*p* < 0.001). These covariates constituted the FM, which demonstrated strong discriminative performance (C-index = 0.838). Assessment of multicollinearity revealed uniformly low variance inflation factors (range, 1.04–1.18), indicating minimal interdependence among predictors and confirming the robustness and stability of the final model (Figure 1). Interaction testing revealed no significant effect of pathological stage on the relationship between the CALM index and OS (*p* for interaction = 0.253), supporting the consistency of its prognostic effect across disease stages.

To further contextualize these findings and compare conventional regression with penalized feature selection, LASSO-penalized Cox regression was performed using 10-fold cross-validation. At the λ value corresponding to the minimum cross-validated partial likelihood deviance, the model selected nine variables with non-zero coefficients: age, ASA-PS, PL, pathological stage, mSLI, the CALM index, smoking status, vascular invasion, and perineural invasion. These variables were subsequently incorporated into a refitted conventional multivariate Cox proportional hazards model. In this model, six covariates—age, ASA-PS, PL, pathological stage, mSLI, and CALM index—remained independently associated with OS, demonstrating substantial concordance with the predictors identified in the primary multivariable Cox regression.

**Figure 2:**
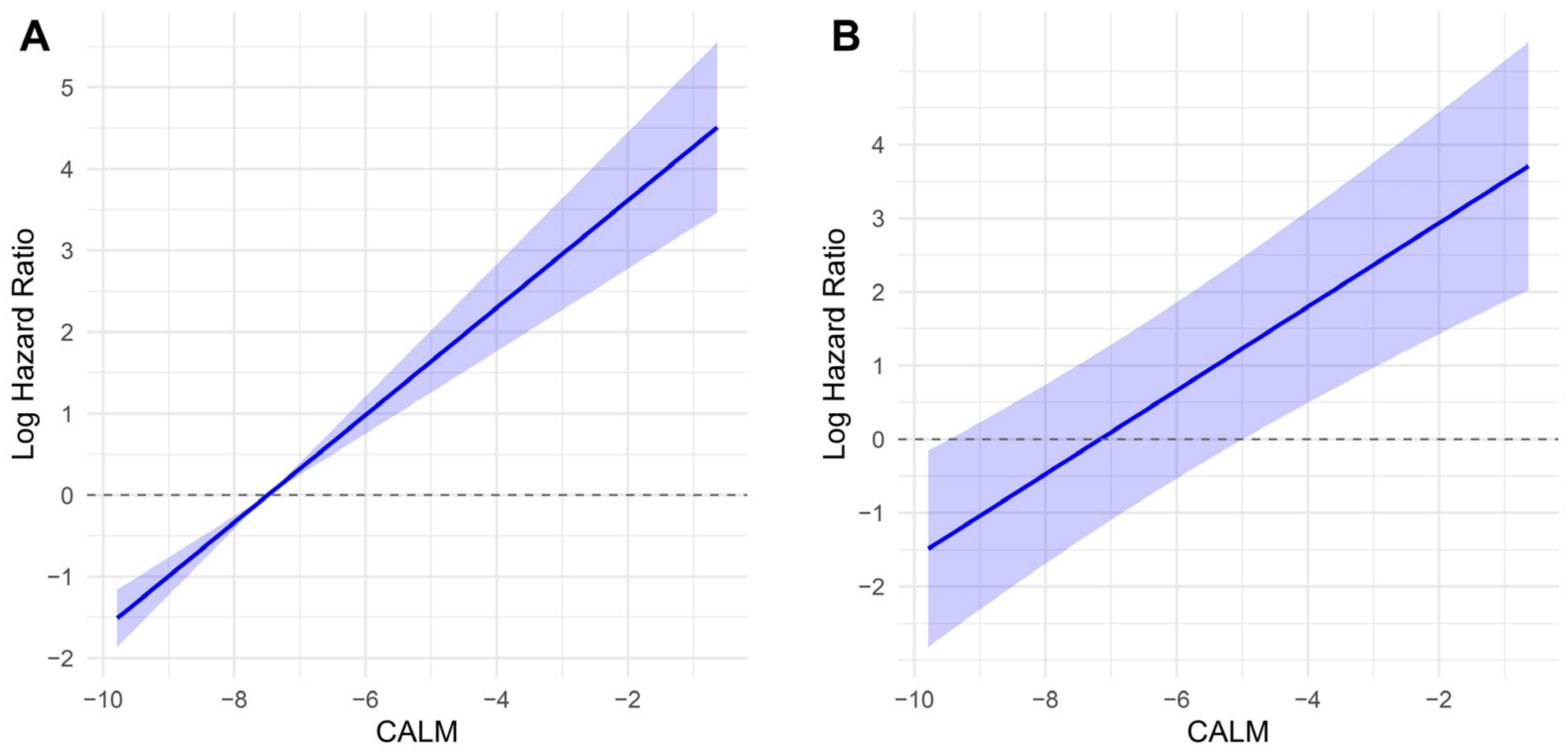
Association between the CALM index and log hazard ratio of death based on Cox regression analysis. (**A**) Univariate model. (**B**) Multivariable model adjusted for age, ASA-PS, PL, pathological stage, and mSLI. Shaded areas indicate 95% confidence intervals.

In the unadjusted analysis, the CALM index exhibited a clear linear relationship with the log-relative hazard of death (Figure 2A). This association retained an approximately linear pattern after multivariate adjustment for established clinical prognostic factors, including age, ASA-PS, PL, pathological stage, and mSLI (Figure 2B). The consistency of this linear pattern in both the crude and adjusted models indicated a dose–response relationship, whereby increasing CALM values corresponded to a steadily rising mortality risk. These findings support the use of the CALM index as a stable and continuous prognostic indicator of OS in patients with NSCLC.

**Figure A1:**
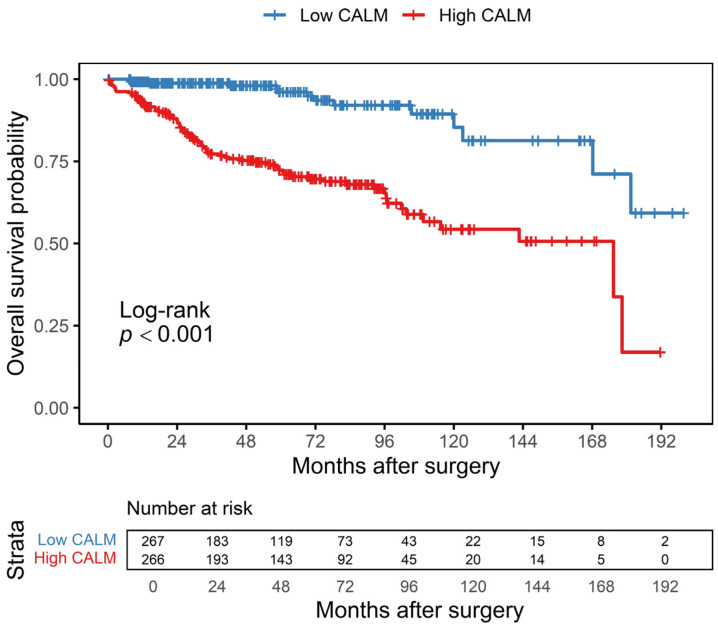
Kaplan–Meier survival curves stratified by CALM index. Overall survival probability comparison between patients with a “Low CALM” index and a “High CALM” index. The threshold for stratification was determined based on the median value of the CALM index. Survival differences were assessed using the log-rank test, with *p* < 0.001 indicating a statistically significant difference in prognosis between the two groups. Number at risk for each group is provided below the plot.

In accordance with our prognostic modeling, Kaplan–Meier analysis demonstrated a significant divergence in survival outcomes between patients stratified by the CALM index median (*p* < 0.001). Patients classified within the ‘Low CALM’ cohort exhibited superior OS compared to those in the ‘High CALM’ group, reinforcing the prognostic utility of the index as a continuous variable even when evaluated through categorical stratification (Figure A1).

Three nested Cox proportional hazards models were compared to determine whether the CALM index provides prognostic information beyond its constituent biomarkers. Likelihood ratio testing showed no significant differences between the CALM-based and component-based models (*p* = 0.688), and the simultaneous inclusion of the CALM and its individual components provided no additional explanatory value (*p* = 0.968). Although discriminative performance was virtually identical across models (C-index: 0.838), the CALM-based model achieved the lowest AIC (889.45), indicating the most parsimonious fit compared with the component-based (893.98) and combined (895.98) models. By distilling the prognostic signal of four distinct biomarkers into a single, highly parsimonious metric, the CALM index eliminates the need for complex multi-variable calculations, thereby facilitating a more efficient and standardized risk-stratification workflow in clinical practice. Collectively, these findings suggest that the CALM index efficiently captures the prognostic information contained within its constituent biomarkers while reducing model complexity, offering a robust and simplified framework for clinical decision-making. Furthermore, because the calculation is mathematically straightforward, it can be seamlessly embedded into electronic medical record systems, allowing for automated, real-time risk assessment without imposing any additional burden on the clinical team.

### 3.3. Biological Architecture of the CALM Index: Relative Contributions and Component Interactions

To elucidate the statistical architecture and underlying biological drivers of the CALM index, we employed a tiered analytical approach. We integrated linear methods—including LASSO and multiple linear regression—with non-linear modeling through XGBoost, supplemented by SHAP for model interpretability. Within the LASSO framework, only four variables—CRP (*β* = 0.0151), albumin (*β* = −1.8098), ALC (*β* = −0.4565), and AMC (*β* = 1.9451)—retained non-zero coefficients, confirming that these biomarkers exclusively define the CALM construct. This finding was corroborated by conventional multiple linear regression analysis, in which all four components remained independently and highly significantly associated with CALM index (all *p* < 0.001), collectively accounting for virtually all observed variance in the index (*R*^2^ = 0.9990).

To independently assess the relative contribution of candidate variables without imposing linear assumptions, we applied an XGBoost model incorporating the full set of candidate clinical and laboratory predictors. Feature importance analysis demonstrated that albumin, CRP, AMC, and ALC were the four most influential variables in predicting risk, whereas all remaining predictors contributed minimally. SHAP-based feature attribution further confirmed the predominance of these four biomarkers in determining CALM values. Based on mean absolute SHAP values, albumin exerted the greatest influence on model predictions (0.534), followed by AMC (0.241), ALC (0.183), and CRP (0.120). The directionality of these effects was biologically coherent: higher albumin and ALC levels were associated with lower predicted CALM values, whereas increasing AMC and CRP levels contributed positively to the index.

Given the dominant contribution of these four biomarkers across both linear and non-linear modeling frameworks, subsequent analyses were directed toward elucidating their individual effects and potential interactions. SHAP dependence analyses were conducted to investigate how these components jointly contribute to prognostic risk and to identify potential effect-modifying relationships. These SHAP dependence plots illustrate the non-linear relationship between individual CALM components and the model-predicted log-hazard (SHAP value). Each panel maps biomarker concentrations along the horizontal axis against their respective SHAP-derived contributions to predicted log-hazard on the vertical axis. The point color (blue gradient) indicates the value of the interacting variable, AMC, while the red curve represents the smoothed global relationship between the biomarker and its prognostic contribution.

**Figure 3:**
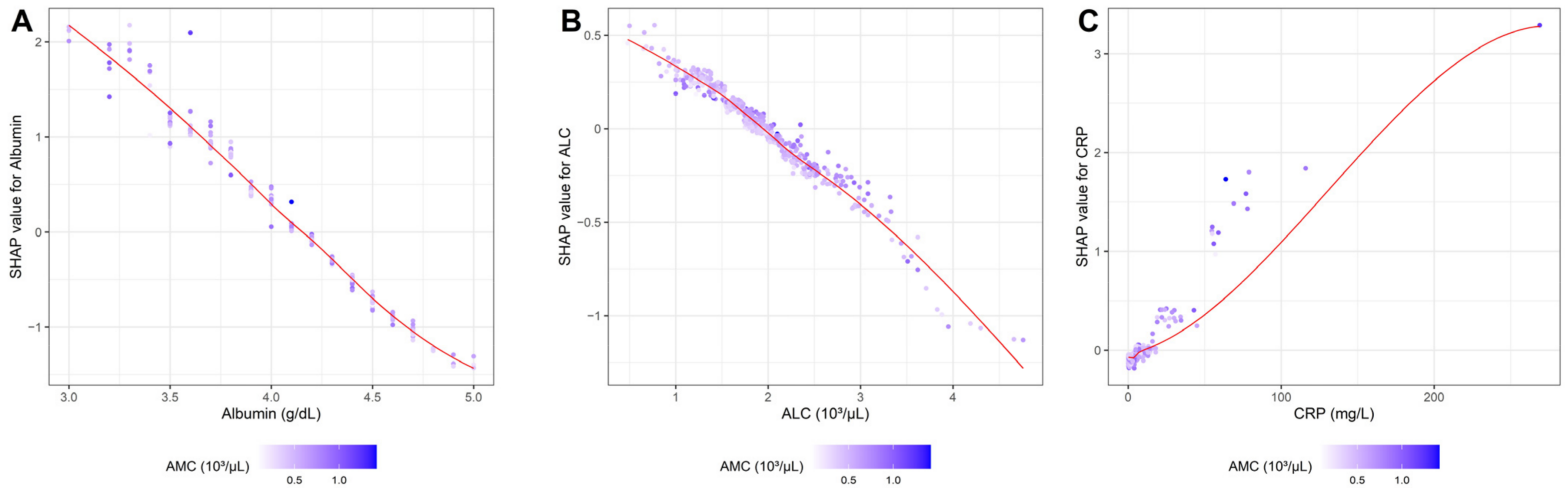
SHAP dependence and interaction plots for the CALM index. In each panel, the *x*-axis displays the biomarker concentration, and the *y*-axis represents the corresponding SHAP contribution. The point color (blue gradient) indicates the value of the interacting variable, AMC, while the red curve represents the smoothed global relationship between the biomarker and its prognostic contribution. (**A**) Albumin dependence plot; (**B**) ALC dependence plot; (**C**) CRP dependence plot.

Regarding the nutritional axis, serum albumin levels showed a robust inverse correlation with clinical risk, underscoring the protein’s role as a primary indicator of physiologic reserve and metabolic stability. SHAP dependence analysis revealed that this relationship is significantly modified by AMC. Notably, as albumin levels decrease toward the hypoalbuminemic range (3.0–4.0 g/dL), higher AMC levels (indicated by darker blue points) are associated with higher SHAP-derived risk estimates than those with lower AMC at the same albumin concentration. This indicates that monocytosis exacerbates the prognostic risk associated with declining nutritional status (Figure 3A).

A similar modifying pattern was observed along the immune axis, represented by ALC. Although higher ALC levels were consistently associated with lower risk, elevated AMC shifted SHAP-derived risk estimates upward across the observed range of lymphocyte counts, indicating that monocytosis diminishes the favorable prognostic impact of preserved lymphocyte levels (Figure 3B).

In contrast, analysis of the inflammatory axis, represented by CRP, demonstrated a positive, non-linear association with risk, characterized by an accelerated increase at higher CRP concentrations. SHAP dependence analysis indicated that this prognostic contribution is modulated by the underlying myeloid inflammatory state (AMC). Specifically, while higher AMC exerted a consistent additive risk-boosting effect across the observed range of CRP (0–260 mg/L), the prognostic impact of increasing CRP appeared attenuated in individuals with the highest baseline myeloid activity. These trends were most robustly observed within the clinically representative range of CRP (<100 mg/L), which accounted for all but two cases in our study population (Figure 3C).

Collectively, these analyses identify AMC as a central, dynamic modifier within the CALM framework. By demonstrating that AMC amplifies hypoalbuminemia-related risk, attenuates the protective effects of lymphocytosis, and exhibits multifaceted interactions with CRP, these findings confirm that the CALM index successfully integrates nutritional, immune, and inflammatory signals into a unified measure of host-related prognostic vulnerability in resected NSCLC.

### 3.4. Assessing the Incremental Prognostic Utility of the CALM Index: A Comparative Framework

To evaluate the clinical benefit of integrating the CALM index into existing diagnostic paradigms, we conducted head-to-head performance comparisons between the FM and two reference models, the BM and the IM. The FM demonstrated markedly superior discriminative performance, achieving a C-index of 0.838 (standard error [SE] = 0.021), exceeding that of the BM (0.690, SE = 0.027). Consistent results were observed for time-integrated discrimination, with an iAUC of 0.799 (SE = 0.018) for FM and 0.655 (SE = 0.025) for BM (both *p* < 0.001). Measures of reclassification further supported the added prognostic value of FM, with IDI values of 0.242 at 3 years and 0.218 at 5 years compared with BM (both *p* < 0.001), indicating sustained improvement in long-term risk prediction.

In contrast to the IM, which yielded a C-index of 0.816 (SE = 0.024) and iAUC of 0.787 (SE = 0.019), FM also showed statistically significant gains in discriminative accuracy (*p* < 0.001 for both metrics). The inclusion of the CALM index resulted in meaningful improvements in risk reclassification, as reflected by the IDI values of 0.056 at 3 years (*p* < 0.001) and 0.039 at 5 years (*p* = 0.024). Together, these findings demonstrate that the incorporation of the CALM index conferred a consistent and incremental enhancement in prognostic performance beyond established clinical predictors (Table 2).

**Figure 4:**
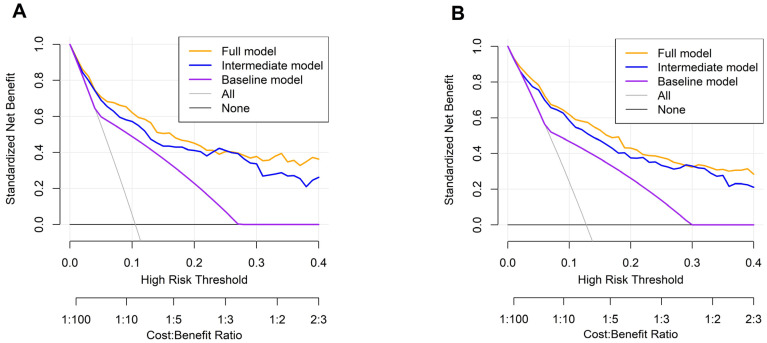
Evaluation of net clinical benefit via decision curve analysis for the full, intermediate, and baseline models in estimating overall survival at 3 years (**A**) and 5 years (**B**).

DCA demonstrated that the FM provided a superior net clinical benefit compared to both the BM and IM across a broad spectrum of threshold probabilities for both 3- and 5-year survival projections. Throughout the clinically relevant decision thresholds, FM maintained higher net benefit values, reflecting a more effective separation of patients at elevated vs. low mortality risk (Figure 4). These results indicated that the utilization of FM has the potential to improve clinical decision-making by reducing overtreatment among low-risk patients, while appropriately identifying individuals who may benefit from intensified postoperative management.

### 3.5. Comparative Prognostic Performance of the CALM Index vs. Established Inflammatory and Nutritional Biomarkers

**Figure 5:**
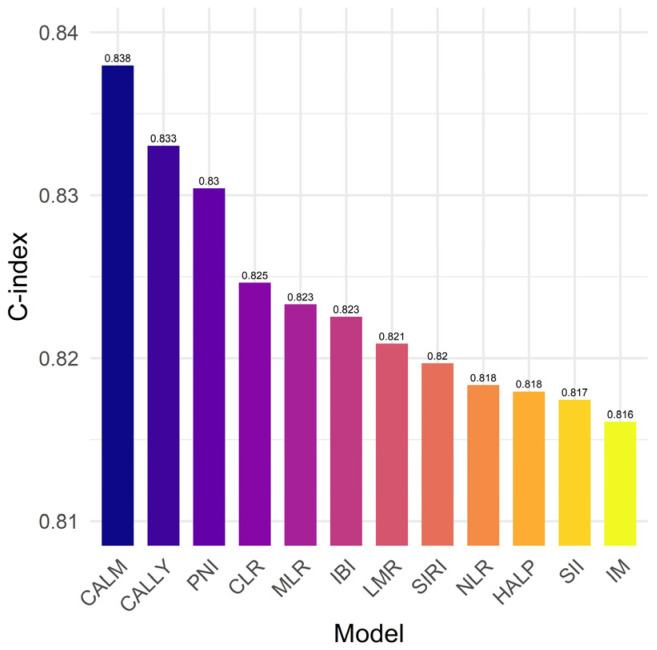
Benchmarking the discriminative performance of the CALM index against established systemic inflammatory and nutritional indices for survival prediction. Markers are ordered by decreasing C-index value: CALM (CRP–albumin–lymphocyte index, 0.838), CALLY (CRP–albumin–lymphocyte, 0.833), PNI (prognostic nutritional index, 0.830), CLR (CRP-to-lymphocyte ratio, 0.825), MLR (monocyte-to-lymphocyte ratio, 0.823), IBI (inflammatory burden index, 0.823), LMR (lymphocyte-to-monocyte ratio, 0.821), SIRI (systemic inflammation response index, 0.820), NLR (neutrophil-to-lymphocyte ratio, 0.818), HALP (hemoglobin–albumin–lymphocyte–platelet score, 0.818), SII (systemic immune-inflammation index, 0.817), and IM (intermediate model, 0.816).

To contextualize the prognostic performance of the CALM index relative to established inflammatory and nutritional biomarkers, we constructed a series of multivariate Cox proportional hazards models in which each biomarker—CALLY index, prognostic nutritional index (PNI), CRP-to-lymphocyte ratio (CLR), monocyte-to-lymphocyte ratio (MLR), inflammatory burden index (IBI), lymphocyte-to-monocyte ratio (LMR), SIRI, neutrophil-to-lymphocyte ratio (NLR), HALP score, and SII—was entered individually into a standardized reference framework corresponding to the IM. The IM incorporated age, ASA-PS, PL, pathological stage, and mSLI, thereby enabling direct and standardized comparisons across biomarkers. Across all the evaluated models, the CALM index demonstrated the strongest discriminative capability (C-index, 0.838). This performance modestly exceeded that of the CALLY index (0.833) and PNI (0.830), whereas other inflammation- and nutrition-based indices, including CLR, MLR, IBI, LMR, SIRI, NLR, HALP, and SII, exhibited comparatively lower discrimination (C-indices, 0.817–0.825) (Figure 5).

### 3.6. Development and Validation of a Full Model-Based Nomogram for 3- and 5-Year Survival Prediction

**Figure 6:**
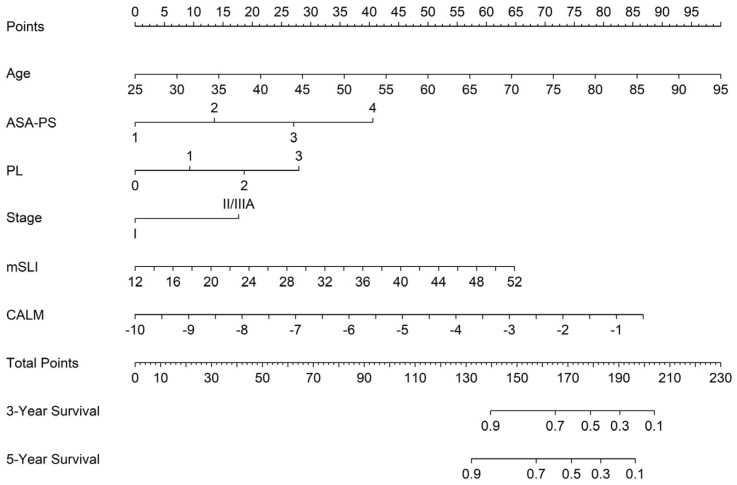
Nomogram plotting the prognostic contribution of clinical variables to 3- and 5-year overall survival in resected stage I–IIIA NSCLC.

A prognostic FM-based nomogram was constructed to generate individualized estimates of 3- and 5-year OS rates. The model incorporated six variables independently associated with survival (age, ASA-PS, PL, pathological stage, mSLI, and the CALM index), integrating the clinical, pathological, and hematologic dimensions of risk. This visual prediction tool holds potential to enable quantitative, patient-specific risk assessment and may facilitate more precise postoperative stratification and informed decision-making regarding surveillance and management (Figure 6).

**Figure 7:**
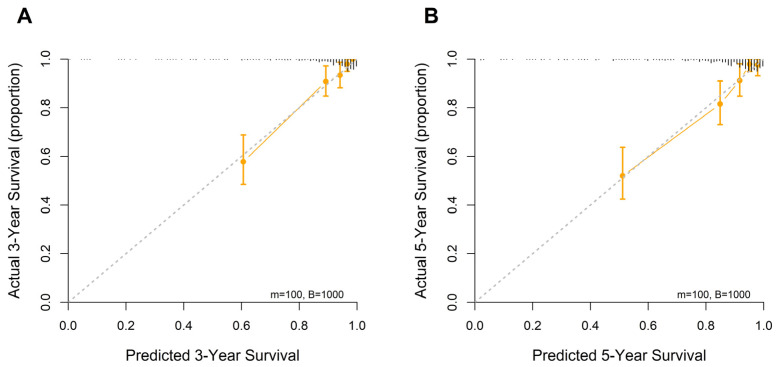
Calibration assessment of the full model for 3-year (**A**) and 5-year (**B**) overall survival estimation.

To verify the prognostic accuracy of the FM, we performed calibration curve analyses at 3 and 5 years, employing internal validation via 1000 bootstrap resamples and an allocation of approximately 100 observations per calibration group. The resulting plots revealed a high degree of concordance, with the observed survival probabilities tracking closely along the 45° diagonal line of perfection. Furthermore, the internal validation process confirmed that the model maintains consistent calibration with minimal optimism, even when stratified across various levels of predicted risk (Figure 7). Collectively, these data confirm the robustness and reliability of the nomogram, validating its utility for generating personalized survival projections in individuals undergoing curative-intent resection for stage I–IIIA NSCLC.

## 4. Discussion

In this study, we comprehensively evaluated the prognostic utility of the CALM index in patients with resected NSCLC using complementary conventional, penalized, and machine learning-based approaches. Across both conventional multivariate Cox regression and LASSO-penalized models, the CALM index consistently emerged as an independent predictor of OS after adjustment for established clinicopathological factors. Furthermore, the incorporation of the CALM index into the full model (FM) significantly improved discrimination, calibration, and clinical net benefit compared with both the baseline model (BM) and the intermediate model (IM). Collectively, these findings support the CALM index as a reproducible and biologically coherent biomarker that provides incremental prognostic information beyond conventional postoperative risk determinants.

Multivariate Cox regression analysis identified age, ASA-PS, pathology, stage, mSLI, and the CALM index as independent predictors of OS. Notably, LASSO-based variable selection followed by refitting retained these same six variables, demonstrating strong concordance with the primary multivariable model. This convergence highlights the consistency and reproducibility of the identified prognostic factors. The persistent selection of the CALM index across both modeling strategies reinforces its prognostic relevance, supporting its potential utility as an integrative biomarker that captures biologically meaningful information beyond traditional clinicopathological features.

Consistent with the results of our multivariable prognostic modeling, the Kaplan–Meier analysis (Figure A1) corroborated the capacity of the CALM index to effectively stratify survival outcomes, confirming a statistically significant prognostic divergence between ‘Low’ and ‘High’ CALM index cohorts.

The retained covariates are clinically and biologically plausible. Age and ASA-PS reflect global physiological reserve and comorbidity burden, and are consistently linked to postoperative outcomes [8,9,10,40]. Pathological stage and PL remain fundamental indicators of tumor aggressiveness and disease extent [7,8,9,10,15]. Furthermore, the inclusion of mSLI, a RBC-derived composite index, highlights the significant contribution of hematologic and systemic factors to patient survival [33,41,42]. Collectively, these findings indicate that outcomes following curative-intent resection are determined not only by anatomical disease burden but also by a patient’s host-related inflammatory, nutritional, and hematologic status.

Importantly, the prognostic association of the CALM index remained consistent across pathological stages, suggesting stable performance irrespective of disease extent. Given the well-recognized heterogeneity in clinical outcomes within TNM stage groups, this finding holds significant practical implications. The CALM index may serve as a complementary tool to refine risk stratification within these stage-defined categories, thereby supporting more individualized postoperative surveillance and tailored adjuvant treatment strategies.

Additional analyses demonstrated that the prognostic information captured by the CALM index was largely equivalent to that contained within its individual components. Models incorporating CRP, albumin, ALC, and AMC separately did not outperform the composite index, and their inclusion alongside the CALM index provided no meaningful incremental prognostic value. Notably, the CALM-based model achieved the lowest AIC, indicating the most favorable balance between model fit and parsimony. These findings suggest that CALM index serves as an efficient integrative biomarker, condensing complementary information related to systemic inflammation, nutritional status, immune competence, and myeloid activation into a single clinically applicable measure of prognostic risk.

Mechanistically, the biological plausibility of the CALM index is supported by the complementary functions of its constituent biomarkers, which collectively reflect four interrelated dimensions of host status: systemic inflammation (CRP), nutritional (albumin), adaptive immune competence (ALC), and myeloid inflammatory activity (AMC). SHAP-based dependence analyses suggest that these components contribute to prognostic risk in a coordinated rather than purely additive manner. In particular, AMC emerged as a key contextual determinant of the prognostic associations of the other CALM components. While albumin and ALC demonstrated inverse associations with risk—consistent with their established roles as markers of physiologic resilience and immune competence—these favorable associations appeared attenuated in the presence of elevated AMC. This suggests that heightened myeloid inflammatory activity may diminish the prognostic benefit typically associated with preserved nutritional and immune status. Conversely, the association between CRP and risk became less pronounced at higher AMC levels, raising the possibility that the incremental prognostic contribution of CRP is attenuated in the setting of pre-existing myeloid activation. Although these observations should not be interpreted as definitive evidence of direct biological interactions, they suggest that the prognostic relevance of individual inflammatory and immune biomarkers is significantly influenced by the broader host inflammatory milieu. Collectively, these findings support the concept that the CALM index captures complementary and interrelated aspects of host biology, thereby providing a more integrated representation of prognostic vulnerability than any single biomarker alone.

From a predictive standpoint, the FM, which incorporates the CALM index alongside established clinical and hematologic covariates, demonstrated superior performance compared with both the BM and IM. These improvements were consistently observed across multiple domains, including discrimination (C-index and iAUC), risk reclassification (IDI), and clinical utility as assessed by decision curve analysis. Although the absolute increase in concordance associated with the CALM index was modest, the improvement was statistically significant and consistent across multiple complementary performance metrics. Notably, even incremental gains in discriminative capacity within an already well-calibrated clinical model can have meaningful clinical impacts, particularly at decision thresholds relevant to adjuvant therapy selection and postoperative surveillance. In this context, the inclusion of the CALM index enhanced the model’s ability to distinguish patients at genuinely elevated risk from those with more favorable prognoses, thereby supporting more appropriate risk-adapted management—minimizing unnecessary interventions in low-risk individuals while facilitating timely treatment for high-risk patients. Collectively, these findings highlight the CALM index as a key integrative component of the FM, providing incremental prognostic information beyond conventional predictors by capturing systemic inflammatory–nutritional status. In doing so, it complements measures of tumor burden and physiological reserve, contributing to a more comprehensive and clinically actionable framework for postoperative risk stratification.

When evaluated against other widely used inflammation- and nutrition-based biomarkers within a uniform prognostic framework, the CALM index demonstrated the highest discriminative performance (C-index = 0.838), outperforming established indices, including the CALLY index, PNI, CLR, MLR, IBI, LMR, SIRI, NLR, HALP, and SII. Notably, while the CALLY index (C-index = 0.833) incorporates CRP, serum albumin, and ALC to reflect systemic inflammation and nutritional status, the superior prognostic performance of the CALM index suggests that the inclusion of the AMC provides critical, incremental prognostic information. Biologically, circulating monocytes serve as precursors to tumor-associated macrophages, which represent a key component of the immunosuppressive myeloid burden within the tumor microenvironment. By facilitating tumor progression, angiogenesis, and resistance to therapy, these cells exert significant influence on clinical outcomes. Thus, the incorporation of AMC likely allows the CALM index to more comprehensively capture the interplay between systemic inflammation and the tumor-promoting myeloid activity that characterizes a more aggressive clinical phenotype.

From an implementation standpoint, the CALM index presents a compelling profile for clinical adoption. Because it relies exclusively on standard, inexpensive laboratory metrics, it avoids the requirement for specialized testing and is therefore highly scalable across varied clinical environments. Once subjected to robust external validation, this index could be seamlessly incorporated into prognostic nomograms or digital decision-support tools. Such integration would provide clinicians with a data-driven foundation to prioritize high-risk patients for enhanced postoperative surveillance or to facilitate the selection of more aggressive, tailored adjuvant therapeutic interventions.

This study has several notable strengths. First, our research offers an in-depth and analytically robust assessment of the CALM index’s utility as a prognostic indicator for patients diagnosed with stage I–IIIA NSCLC who have undergone curative-intent surgery. To the best of our knowledge, this work represents the inaugural investigation into the predictive efficacy of this specific composite index—which synthesizes CRP, albumin, ALC, and AMC—within a surgically managed NSCLC cohort, thereby filling a significant void in current postoperative risk-assessment strategies. This analysis leveraged a well-characterized patient cohort featuring standardized staging protocols and extended follow-up; additionally, we maintained continuous predictors in their native formats to circumvent the information loss typically associated with arbitrary dichotomization. Second, this study is strengthened by the rigorous use of complementary analytical frameworks, including conventional multivariable Cox regression, LASSO-penalized feature selection, and machine learning-based interpretability. Across these approaches, the CALM index was consistently identified as an independent predictor of OS alongside established clinicopathological factors, contributing to a final prognostic model with excellent discriminative performance and minimal multicollinearity. The high concordance between the predictors selected using standard and penalized regression methods supports the robustness, stability, and generalizability of the findings. Third, we conducted an exhaustive evaluation of model performance via discrimination, calibration, reclassification, and DCA, with internal bootstrap validation confirming predictive stability. Importantly, the prognostic value of the CALM index was continuous and approximately linear across both unadjusted and fully adjusted models, supporting its use without arbitrary categorization and maximizing prognostic resolution. Finally, incorporation of the CALM index into multivariable models yielded consistent, meaningful improvements in discrimination and risk reclassification, while significantly enhancing the net clinical benefit compared to both the BM and IM, as well as compared with a broad spectrum of established inflammatory and nutritional biomarkers within a uniform comparative framework. Given that the CALM index is derived from routinely available, low-cost laboratory parameters without the need for specialized testing, it has strong translational potential and practical scalability across diverse healthcare settings. Nevertheless, external validation in independent and heterogeneous cohorts remains necessary to confirm its generalizability and clinical applicability.

Nevertheless, this study has several limitations. First, our retrospective, single-center design potentially introduces selection bias and limits the generalizability of our findings. The study population was overwhelmingly East Asian (98.1%), which may reflect unique genetic, environmental, and dietary factors not representative of Western or other ethnically diverse cohorts. While this uniformity allowed for the collection of high-quality, standardized clinical data—essential for the precise development of the CALM index—it necessitates caution in extrapolating these results to broader populations. Furthermore, because the CALM index’s weighted formula was derived from internal Cox regression coefficients optimized for our institutional cohort, these fixed weightings may not be directly generalizable to other settings. We therefore advise against the direct application of this formula in clinical practice without prior external validation and re-calibration. To address these limitations, we propose a two-phase verification scheme: first, a retrospective validation in an independent, international cohort to assess the reproducibility of our coefficients across different environments; second, a prospective, multi-institutional trial that incorporates serial CALM measurements, molecular profiling, and standardized treatment protocols. Such investigations are essential to confirm the robustness of the CALM index, refine its prognostic weightings across diverse ethnic populations, and definitively establish its utility within routine postoperative risk stratification and clinical decision-making frameworks [43,44].

Second, although 73.0% of our cohort presented with stage I disease, we systematically incorporated pathological stage as a covariate into all prognostic models to mitigate potential stage-related confounding and ensure the independent predictive value of the CALM index. Third, the analysis relied exclusively on preoperative baseline laboratory values, precluding evaluation of temporal fluctuations. While longitudinal assessment may provide additional prognostic information, postoperative measurements can be influenced by adjuvant therapy, treatment-related toxicities, or perioperative stress, which complicates interpretation. Moreover, individual components of the CALM index may be affected by non-malignancy-related conditions, including acute infection or chronic autoimmune diseases. To minimize such bias, patients with active systemic inflammatory conditions were excluded, and laboratory measurements were restricted to those obtained within 7 days before surgery. Fourth, the study’s primary endpoint was OS rather than recurrence-free or cancer-specific survival. In this retrospective cohort, complete data to reliably ascertain recurrence events were not uniformly available. Consequently, competing non-cancer deaths may have influenced our findings, particularly among older patients or those with a significant comorbidity burden; however, OS remains the most objective and validated outcome measure, minimizing the ascertainment bias associated with other endpoints.

## 5. Conclusions

In summary, the CALM index provides a clinically actionable and biologically coherent measure of prognostic risk, demonstrating independent predictive value for survival outcomes in surgically treated NSCLC cohorts. Across complementary analytic frameworks—including both conventional and penalized regression approaches—the CALM index demonstrated consistent prognostic performance and provided incremental predictive and clinical utility beyond established clinicopathological factors and commonly used inflammatory or nutritional indices. By synthesizing metrics of systemic inflammation, nutritional reserves, and immune cell dynamics, this index provides an efficient, low-cost instrument for refined postoperative risk assessment. Once verified through external validation in heterogeneous patient populations, the CALM index holds potential to guide personalized follow-up and optimize perioperative decision-making, ultimately improving the delivery of patient-centered therapeutic interventions.

## Figures and Tables

**Table 1:** Clinicopathological characteristics of the patients.

Variables	*n* (%) or Median (IQR)	Variables	*n* (%) or Median (IQR)
Age, years	69 (12)	Vascular invasion	
Sex		Yes	29 (5.4%)
Men	310 (58.2%)	No	504 (94.6%)
Women	223 (41.8%)	Perineural invasion	
Smoking		Yes	8 (1.5%)
Current/Past	214 (40.2%)	No	525 (98.5%)
Never	319 (59.8%)	Pathological stage	
Alcohol consumption		IA/IB	389 (73.0%)
Yes	135 (25.3%)	IIA/IIB/IIIA	144 (27.0%)
No	398 (74.7%)	Adjuvant therapy	
ASA-PS		Yes	121 (22.7%)
1/2	438 (82.2%)	No	412 (77.3%)
3/4	95 (17.8%)	Total protein, g/dL	7.1 (0.7)
BMI, kg/m^2^	23.9 (4.2)	Albumin, g/dL	4.2 (0.4)
Resection		Total bilirubin, mg/dL	0.5 (0.2)
Sublobar resection	194 (36.4%)	AST, U/L	22 (8)
Lobectomy	326 (61.2%)	ALT, U/L	17 (11)
Bilobectomy	6 (1.1%)	CRP, mg/L	1.0 (2.4)
Pneumonectomy	7 (1.3%)	WBC, ×10^3^/μL	6.3 (2.2)
Histological subtype		ANC, ×10^3^/μL	3.6 (1.7)
Squamous	118 (22.1%)	ALC, ×10^3^/μL	1.8 (0.7)
Non-squamous	415 (77.9%)	AMC, ×10^3^/μL	0.5 (0.2)
Tumor size, cm	2.5 (1.8)	Hemoglobin, g/dL	13.3 (2.0)
Pleural invasion (PL)		MCV, fL	91.8 (5.6)
PL0	425 (79.7%)	MCH, pg	30.9 (2.1)
PL ≥ 1	108 (20.3%)	MCHC, g/dL	33.7 (1.2)
Lymphatic invasion		mSLI	26.1 (4.7)
Yes	66 (12.4%)	Platelet, ×10^3^/μL	235 (76)
No	467 (87.6%)		

ASA-PS, American Society of Anesthesiologists physical status; BMI, body mass index; IQR, interquartile range. Hematological indices: ANC, absolute neutrophil count; ALC, absolute lymphocyte count; AMC, absolute monocyte count; MCV, mean corpuscular volume; MCH, mean corpuscular hemoglobin; MCHC, mean corpuscular hemoglobin concentration; mSLI, modified Shine–Lal index; WBC, white blood cell count. Biochemical markers: ALT, alanine aminotransferase; AST, aspartate aminotransferase; CRP, C-reactive protein.

**Table 2:** Model comparison for survival prediction.

Metrics	FM vs. BM		FM vs. IM	
	Improvement	*p*-Value	Improvement	*p*-Value
C-index	0.151 (0.022)	<0.001	0.021 (0.010)	<0.001
iAUC	0.144 (0.009)	<0.001	0.014 (0.003)	<0.001
IDI at 3 years	0.242 (0.046)	<0.001	0.056 (0.024)	<0.001
IDI at 5 years	0.218 (0.041)	<0.001	0.039 (0.021)	0.024

Values in parentheses indicate standard errors. BM, baseline model; C-index, Harrell’s concordance index; FM, full model; iAUC, integrated area under the curve; IDI, integrated discrimination improvement; IM, intermediate model.

## Data Availability

The datasets presented in this study are available upon request from the corresponding author due to ethical reasons.
